# Prevalence of discontinuation of contraceptives due to failure among women aged 14 to 49 years in Uganda: a nation wide cross-sectional survey

**DOI:** 10.1186/s40834-022-00210-y

**Published:** 2023-02-09

**Authors:** Ruth Ketty Kisuza, Saviour Kicaber, Derrick Bary Abila, Felix Bongomin, Christopher Orach Garimoi

**Affiliations:** 1 College of Health Sciences, Makerere University, P.O Box 7072, Kampala, Uganda; 2 Department of Medical Microbiology and Immunology, Faculty of Medicine, Gulu University, Gulu, Uganda; 3 School of Public Health, Makerere University, Kampala, Uganda

**Keywords:** Contraceptive failure, Contraception Discontinuation, Uganda, Demographic Health Survey

## Abstract

**Background:**

Sustained motivation is essential for effective use of contraceptive methods by women in low- and middle-income countries as many women are likely to abandon contraceptives, especially when they continually experience episodes of failure. We aimed to determine the prevalence of discontinuation of contraceptives due to failure and its associated factors among Ugandan women aged 14–49 years.

**Methods:**

A cross sectional study was conducted using the UDHS 2016 data. Multi stage stratified sampling was used to select participants. All eligible women aged 15 to 49 years at the time of the survey were enrolled. Bivariable and Multivariable logistic regression analysis were used to determine the factors associated with contraceptive failure. All analysis were done using Stata version 13. Contraceptive failure (getting pregnant while on contraceptives) within five years preceding the survey was the dependent variable.

**Results:**

A total of 9061 women were included in the study. The overall prevalence of contraceptive failure was 5.6% [*n* = 506, 95% CI: 5.1–6.1] and was higher (6.2%) among women aged 20–29 years or had completed secondary education (6.1%). Having informed choice on contraceptives [aOR = 0.59, 95% CI: 0.49 – 0.72] and older age [aOR = 0.46, 95% CI: 0.24–0.89] were associated with lower odds of contraceptive failure.

**Conclusion:**

The burden of contraceptive failure among women of reproductive age in Uganda is substantial and significantly varied by women's age, level of education, exposure to the internet, mass media, and informed choice. These findings highlight the need for improved counseling services and contraceptive quality to help women and couples use methods correctly and consistently.

## Background

Improving access to family planning (FP) services is fundamental to achieving the Sustainable Development Goals (SDGs) because it is strongly related with women’s and children's health, poverty reduction, education, gender equality, and human rights [1]. Access to family planning contributes up to a 44% reduction in maternal deaths [2], as most unplanned pregnancies and abortions occur in women who were either not using contraception or not using it consistently [3–6].Greater access to contraception and more consistent use are crucial in reducing unplanned pregnancies and abortions [7, 8].

The reasons for discontinuation of contraceptives can be grouped by whether they represent discontinuations due to reduced need (not in need) or discontinuations while women are presumably still exposed to the risk of pregnancy and do not want to become pregnant (in need) [9]. Among the not in-use discontinuations, wanting to become pregnant is the common reason while becoming pregnant yet using contraceptives (i.e. contraceptive failure) and side effects are the common reasons for in-need discontinuations [9].

Discontinuation of contraceptives for reasons other than the desire for pregnancy is problematic because of it’s association with several adverse reproductive health outcomes [10]. In countries with moderate to high contraceptive prevalence, most unintended pregnancies result from contraceptive failure [4, 11, 12]. Several studies have associated contraceptive discontinuation for reasons other than the desire to become pregnant with an unmet need for contraception and induced abortion [9, 13, 14]. Unintended pregnancies have been associated with an increased risk of maternal morbidity, health behaviors during pregnancy that are associated with adverse maternal health, and adverse fetal, infant, and child health outcomes [15]. Additionally, unintended pregnancies have negative psychological effects on women and their children [16].

In developing countries, 74 million unintended pregnancies occur annually, and nearly a third 30%, are due to contraceptive failure among women using some type of contraceptive method (whether traditional or modern).This includes both method-related failures (i.e., failure of a method to work as expected) and user-related failures (i.e., failure due to incorrect or inconsistent use of a method) [17].

About 44% of pregnancies in Uganda are unintended [18], with the occurrence of unsafe abortions estimated at 62% per 1000 women aged 15-49 years [19]. These undesirable maternal and child health outcomes associated with a high total fertility rate of 6.2 [20]could be substantially reduced by meeting the family planning needs of women in developing countries [21]. Moreover, a number of studies in Uganda have reported several obstacles to continuous contraceptive use among women of reproductive age, among which are method-related failures [8, 22–24].

Sustained motivation is essential for the effective use of contraceptive methods by women in low- and middle-income countries, as many women are likely to abandon the use of contraceptives, especially when they continually experience episodes of failure. A study exploring the predictors of contraceptive adherence among women seeking family planning services at a Reproductive Health Clinic in Uganda reported that lower educational level, lower self-efficacy, and lack of male partner support were predictive of reporting short birth intervals of less than 2 years [25]. Most Studies in Uganda have reported on the association between contraceptive use and discontinuation as a result of side effects, partner influence, and service quality, with limited literature on contraceptive failure [26–28]. Assessing contraceptive failure among demographic and socio-economic groups is important to inform efforts to improve contraceptive information, services and use, and to minimize contraceptive failures. Additionally, detailed information on contraceptive discontinuation due to unintended pregnancy is critical to inform improvements in the provision of contraceptive information, supplies, and services, which can help women and couples use methods correctly and consistently.

This study aimed to determine the prevalence of discontinuation of contraceptives due to failure among women aged 14–49 years using the 2016 UDHS data.

## Methods

### Study sampling and participants

This was a cross-sectional study. We analyzed data collected during the UDHS conducted in 2016. "The Demographic and Health Surveys (DHS) are internationally comparable household surveys that collect information on demographic, socioeconomic, and health-related variables among nationally representative samples of households in developing countries. The DHS employs a two-stage sample design, with the first stage involving cluster selection consisting of enumeration areas (EAs). The second stage involves the systematic sampling of households in all the selected EAs” [29]. In this study, we used the women's recode file, which included women aged 15 to 49 years at the time of the survey. Out of the 18,505 women included in the UDHS women recode file, we included only women who had ever or were current users of contraceptives within the 5 year period preceding the survey to our study giving a total of 9,061 study participants.

## Study variables

### Dependent variable

Discontinuation of contraceptive use due to failure within a five-year period preceding the survey was the dependent variable. The DHS does not have a variable that records the discontinuation of contraceptive use due to failure. This was derived from the variable v360 which records the various reasons for discontinuation i.e., why did you stop using (Method)? The options/responses were 1) became pregnant, 2) wanted to become pregnant, 3) husband disapproved and 4) side effects among others. In this study, a woman becoming pregnant while on contraceptives was termed as a method failure and was coded as the outcome variable, while discontinuation due to another reason was coded for those who did not have the outcome of interest.

### Independent variables

We included possible determinants of contraceptive use and discontinuation based on the available literature [7, 27, 30, 31]. Twenty-one (21) variables were considered and grouped as follows. Demographic factors which included (1) age in years (categorized as > 20, 20–29, 30–39, and 40–49), and the number of children delivered (categorized as none, 1,2,3,4 and 5 or more). Socio-economic factors which include (1) type of place of residence (rural vs. urban), (2) wealth index (categorized as lowest, second, middle, fourth, and highest), (3) Women's education attainment (Categorized as no formal education, primary education, secondary education, and higher), (4) phone ownership (yes vs. no), (6) internet use (categorized as Never, yes-last 12 months and yes –before last 12 months), (7) region of residence (Categorized as Northern, Eastern, Western and Southern), (8) work status (categorized as not currently employed and currently employed), (9) received information on family planning from mass media like radio, telephone, television and newspaper or magazine, (10) heard of family planning from field worker (Yes vs. no), and (11) heard of family planning from a healthcare facility (yes vs no). Cultural factors like 1) women's participation in decision-making for using contraceptives (categorized as woman only, husband only, joint decision and other), and informed choice (yes vs. no),Lastly, we also included the variable on the method of contraception (Modern vs. traditional) that was discontinued over the 5-year period preceding the survey.

Informed choice was a new variable created from five variables measured in the UDHS. These measured whether the woman was (1) told how about the side effects of contraceptives, (2) told about the side effects of contraceptives by a health or family planning worker, (3) told how to deal with side effects, (4) told about other family planning methods, and (5) told about other family planning methods by health or family planning worker. A woman was considered to have an informed choice if they responded with yes to any of the five variables.

## Statistical analysis

The data used were obtained after receiving permission from the DHS program website. The dataset for the Uganda DHS 2016 was then downloaded from the DHS program website. The women's recode file, readable by Stata version 15 was selected for use.

To calculate the prevalence of discontinuation due to contraceptive failure, we created a new variable for which the denominator would be the total of the women who were currently using contraceptives at time of the survey interview and those who were not using at the time of the survey because they had discontinued.

All the analysis in this study was performed in Stata 13 (StataCorp LP 2013). Weighting was performed for all the descriptive statistics and for regression analysis.

For the categorical variables, we summarized them as weighted proportions. Variables that were numeric like age, were summarized as weighted means reporting standard deviations and 95% confidence intervals.

Bivariable logistic regression was used to determine the association between discontinuation due to contraceptive failure and the independent variables. From this, independent variables with *p-values* less than 0.2 were subjected to a test to assess for multi-collinearity using “variance inflation factor (VIF)” on Stata software. There was no multi-collinearity as the VIF were less than 3 for all the included variables.

Multivariable logistic regression was used, including the variables that had a *p*-value of less than 0.200 and variables that have been shown by literature to influence failure related contraceptive discontinuations [25, 32] i.e., age group, number of children a woman has, frequency of using the internet last month, heard about family planning from mass media, education and informed choice among others. We reported crude odds ratios (bi-variable), adjusted odds ratios (multi-variable), *p-values*, and 95% confidence intervals.

## Results

### Characteristics of the women

Table 1 shows the socio-demographic characteristics of the sample of women aged 15–49 who were current users/had ever used a contraceptive method within five years before the 2016 UDHS (*n* = 9061). The majority of the women (46.4%, *n* = 4204) were between age 20–29 years, and only 7.5% of the women had no formal education. Majority (80.3%) of the women were employed, and about 70.7% lived in rural areas of Uganda. Majority of women who used contraceptives had never been exposed to the internet in the last 12 months (89.0%, *n* = 8067). Additionally, 56.8% (*n* = 5153) of the women who used contraceptives owned a mobile phone. Among cultural factors, about two-thirds (61.2%) of the women made a joint decision with their partners on contraceptive use. 41.8% (*n* = 3788) of the women using contraceptives made an informed choice, while 58.2% (*n* = 5273) of the women did not make an informed choice. The most common methods used and discontinued in the past 5 years were injectable (54.8%), followed by implants (10.5%).

Current users or ever used contraceptives in past 5 years (9061).

**Table 1:** Distribution of method related, demographic, socio economic and cultural characteristics of Ugandan women aged 15–49 years by status of contraceptive use

	**Frequency (%)**
**Demographic factors**	
**Age**	
Less than 20 years	691(7.6)
20 to 29 years	4204(46.4)
30 to 39 years	2924(32.3)
40 to 49 years	1242(13.7)
**Number of children ever born**	
None	678(7.5)
One	1298(14.3)
Two	1489(16.4)
Three	1312(14.5)
Four	1164(12.8)
Five or more	3119(34.4)
**Socioeconomic factors**	
**Internet use in the past year**	
Never	8067(89.0)
Yes, last 12 months	873(9.6)
Yes, before last 12 months	120(1.3)
**Frequency of using internet last month**	
Not at all	8230(90.8)
Less than once a week	119(1.3)
At least once a week	285(3.1)
Almost every day	426(4.7)
**Mobile phone ownership**	
No	3907(43.1)
Yes	5153(56.9)
**Women's education attainment**	
No formal education	678(7.5)
Primary education	5075(56.0)
Secondary education	2421(26.7)
Higher	887(9.8)
**Currently/formerly/never in union**	
Never in union	863(9.5)
Currently in union/living with a man	6939(76.6)
Formerly in union/living with a man	1259(13.9)
**Work status**	
Not currently employed	1784(19.7)
Currently employed	7276(80.3)
**Place of residence**	
Urban	2650(29.2)
Rural	6411(70.8)
**Region of Uganda**	
Central	3019(33.3)
Eastern	2420(26.7)
Western	1350(14.9)
Southern	2273(25.1)
**Household wealth index quintile**	
Lowest	1205(13.3)
Second	1601(17.7)
Middle	1725(19.0)
Fourth	1956(21.6)
Highest	2573(28.4)
**Heard about FP from any source**	
No	1717(18.9)
Yes	7344(81.1)
**Heard about FP from field health worker**	
No	8271(91.3)
Yes	790(8.7)
**Heard about FP at health facility**	
No	6147(67.8)
Yes	2914(32.2)
**Heard about family planning on mass media**	
No	2401(26.5)
Yes	6659(73.5)
**Heard about FP on radio**	
No	2761(30.5)
Yes	6299(69.5)
**Heard about FP on Television**	
No	6979(77.0)
Yes	2082(23.0)
**Heard about FP on Newspaper/magazine**	
No	7938(87.6)
Yes	1122(12.4)
**Heard about FP on phone**	
No	8709(96.1)
Yes	352(3.9)
**Cultural factors**	
**Women participation in decision-making for using contraceptives**	
Mainly woman	1340(30.6)
Mainly husband/partner	312(7.1)
Joint decision	2710(61.2)
Other	11(0.3)
**Informed choice**	
No	5273(58.2)
Yes	3788(41.8)
**Method-related factors**	
**Contraceptive method used**	
Pill	553(9.6)
Intra-Uterine Device (IUD)	161(2.8)
Injectable	3150(54.8)
Male condom	444(7.7)
Male sterilization	1(0.0)
Periodic abstinence	188(3.3)
Withdraw	371(6.5)
Other traditional	42(0.7)
Implants/Norplant	605(10.5)
Lactational amenorrhea (lam)	155(2.7)
Female condom	2(0.0)
Emergency contraception	22(0.4)
Other modern method	15(0.3)
Standard days method (sdm)	35(0.6)

### Prevalence of discontinuation due to contraceptive failure

As shown in Table 2, the overall prevalence of contraceptive failure among Ugandan women aged 15–49 years during the 5-year period by demographic, socio-economic, and cultural factors was 5.6% (*n* = 506, 95% CI: 5.1—6.1). Among demographic factors, Contraceptive failure was prevalent in women aged 20–29 years (6.2%) and in those with five or more children (6.4%). For socioeconomic factors, the highest prevalence rates were seen in women who used the internet almost every day in the one month (7.8%, *n* = 426), owned a mobile phone (5.7%, *n* = 294), completed higher education (7.0%, *n* = 63), unemployed (5.8%, *n* = 104) and lived in a rural area (5.7%, *n* = 364).

**Table 2:** Prevalence of discontinuation due to contraceptive failure among Ugandan women aged 15–49 years during a five-year period by demographic, socioeconomic, and cultural factors

Demographic characteristics	Number using contraceptives	Freq of discontinuation. (%)
**Demographic factors**		
**Age**		
Less than 20 years	691	19(2.8)
20 to 29 years	4204	261(6.2)
30 to 39 years	2924	171(5.9)
40 to 49 years	1242	54(4.3)
**Total children born**		
None	678	27(4.0)
One	1298	58(4.5)
Two	1489	73(4.9)
Three	1312	77(5.9)
Four	1164	73(6.3)
Five or more	3119	198(6.4)
**Socioeconomic factors**		
**Internet use in the past year**		
Never	8067	440(5.5)
Yes, last 12 months	873	57(6.6)
Yes, before last 12 months	120	8(6.8)
**Frequency of using internet last month**		
Not at all	8230	450(5.5)
Less than once a week	119	6(4.7)
At least once a week	285	17(5.9)
Almost every day	426	33(7.8)
**Mobile phone ownership**		
No	3907	211(5.4)
Yes	5153	295(5.7)
**Heard about family planning from any source**		
No	1717	68(4.0)
Yes	7344	437(6.0)
**Heard about FP from field health worker**		
No	7811	460(5.6)
Yes	790	46(5.8)
**Heard about FP at health facility**		
No	6147	342(5.6)
Yes	2914	164(5.6)
**Heard about FP on radio**		
No	2761	137(5.0)
Yes	6299	368(5.8)
**Heard about FP on Television**		
No	6979	372(5.3)
Yes	2082	133(6.4)
**Heard about FP on Newspaper/magazine**		
No	7938	417(5.2)
Yes	1122	89(7.9)
**Heard about FP on Phone**		
No	8709	474(5.4)
Yes	352	32(9.1)
**Mass media**		
No	2401	109(4.6)
Yes	6659	396(5.9)
**Education**		
No education	678	35(5.1)
Primary	5075	260(5.1)
Secondary	2421	148(6.1)
Higher	887	63(7.1)
**Currently/formerly/never in union**		
Never in union	863	24(2.7)
Currently in union/living with a man	6939	431(6.2)
Formerly in union/living with a man	1259	51(4.0)
**Work status**		
Not currently employed	1784	104(5.8)
Currently employed	7277	402(5.5)
**Place of residence**		
Urban	2650	140(5.3)
Rural	6411	365(5.7)
**Household wealth index**		
Poorest	1205	64(5.3)
Poorer	1601	85(5.3)
Middle	1725	97(5.6)
Richer	1956	116(5.9)
Richest	2573	144(5.6)
**Cultural factors**		
**Women participation in decision-making for using contraceptives**		
Mainly woman	1340	64(4.8)
Mainly husband/partner	312	9(3.0)
Joint decision	2710	134(5.0)
**Informed choice**		
No	5273	338(6.4)
Yes	3788	168(4.4)

Among cultural factors, women who had a joint decision for contraceptive use with their partner had the highest failure of the 5.0% (*n* = 134). Women who made an informed choice on contraceptive use experienced low contraceptive failure (4.4%, *n* = 168) as compared to those who didn't make an informed choice (6.4%, *n* = 338).

Table 3 shows the contraceptive failure in Uganda by the method used. Generally, traditional methods had the highest failure. Of this, withdrawal (36.3%, *n* = 134) was the single most common reason for failure. For modern contraceptive methods, short-term methods like emergency contraception (20.9%, *n* = 4) and pills (10.7%, *n* = 59) had higher failure when compared to long- term modern contraceptives like IUD (1.6%, *n* = 3) and implants (2.6%, *n* = 15) which had a very low failure.

**Table 3:** Prevalence of Contraceptive failure in Uganda by methods used among Ugandan women aged 15–49 years

Contraceptive method discontinued	Number who used the method	Number that discontinued due to failure. (%)
**Modern Methods**		
Pill	553	59(10.7)
IUD	161	3(1.6)
Injectable	3150	140(4.4)
Male condom	444	31(6.9)
Male sterilization	1	0(0.0)
Implants/Norplant	605	16(2.6)
Lactational amenorrhea (lam)	155	20(12.7)
Female condom	2	1(45.8)
Emergency contraception	22	4(20.9)
Other modern method	15	2(13.4)
**Traditional methods**		
Periodic abstinence	188	65(34.9)
Withdraw	371	134(36.3)
Other traditional	42	19(44.8)
Standard days method (sdm)	35	12(34.2)

### Factors associated with contraceptive failure

Results from Bivariable analysis with their respective crudes odds ratios are detailed in Table 4. From multivariable analysis, women aged 40 to 49 years [Adjusted Odds ratio (aOR), 0.46; 95% CI: 0.24 – 0.89] were less likely to discontinue contraceptives due to failure compared to those aged less than 20 years.

**Table 4:** Factors associated with contraceptive failure among Ugandan women aged 15–49 years in Uganda

	**Bivariable analysis**		**Multivariable analysis**	
**Demographic characteristics**	**cOR** **(95% CI)**	***P*—value**	**aOR (95% CI)**	***P*—value**
**Age of the woman**				
Less than 20 years	1		1	
20 to 29 years	2.16 (1.33 – 3.49)	0.002	1.35 (0.79 – 2.29)	0.272
30 to 39 years	2.13 (1.31 – 3.47)	0.002	0.82 (0.45 – 1.49)	0.516
40 to 49 years	1.38 (0.80 – 2.38)	0.250	**0.45 (0.24 – 0.87)**	**0.018**
**Number of children ever born**				
No child	1		1	
One child	1.58 (0.93 – 2.66)	0.089	1.43 (0.78 – 2.62)	0.244
Two children	1.45 (0.87 – 2.44)	0.157	1.30 (0.68 – 2.46)	0.427
Three children	1.80 (1.08 – 3.01)	0.024	1.78 (0.93 – 3.43)	0.084
Four children	1.90 (1.13 – 3.18)	0.015	**2.21 (1.13 – 4.32)**	**0.021**
Five or more children	2.16 (1.35 – 3.47)	0.001	**3.67 (1.87 – 7.23)**	** < 0.001**
**Socioeconomic factors**				
**Internet use in the past year**				
Never	1			
Yes, last 12 months	1.08 (0.78 – 1.49)	0.637	**-**	**-**
Yes, before last 12 months	1.42 (0.70 – 2.88)	0.329	**-**	**-**
**Frequency of using internet last month**				
Not at all	1		1	
Less than once a week	0.73 (0.28 – 1.88)	0.512	0.81 (0.31 – 2.13)	0.675
At least once a week	1.46 (0.89 – 2.40)	0.136	1.54 (0.89 – 2.65)	0.120
Almost every day	1.01 (0.64 – 1.59)	0.971	1.12 (0.66 – 1.88)	0.684
**Mobile phone ownership**				
No	1			
Yes	0.99 (0.82 – 1.19)	0.918	**-**	**-**
**Woman’s Education**				
No education	1		1	
Primary education	1.03 (0.72 – 1.47)	0.887	1.10 (0.76 – 1.58)	0.627
Secondary education	1.18 (0.81 – 1.73)	0.393	**1.51 (1.01 – 2.27)**	**0.047**
Higher	1.21 (0.77 – 1.89)	0.416	**1.77 (1.05 – 2.98)**	**0.032**
**Currently/formerly/never in union**				
Never in union	1		1	
Currently in union/living with a man	2.15 (1.41 -3.28)	< 0.001	**1.75 (1.03 –2.99)**	**0.038**
Formerly in union/living with a man	1.39 (0.836 – 2.30)	0.205	1.19 (0.65 – 2.16)	0.578
**Work status**				
Not currently employed	1			
Currently employed	1.02 (0.80 – 1.30)	0.849	**-**	**-**
**Place of residence**				
Urban	1			
Rural	1.03 (0.83 – 1.27)	0.803	**-**	**-**
**Household wealth index quintile**				
Lowest	1			
Second	1.06 (0.78 – 1.45)	0.712	**-**	**-**
Middle	0.98 (0.71 – 1.35)	0.901	**-**	**-**
Fourth	1/.05 (0.77 – 1.43)	0.747	**-**	**-**
Highest	0.95 (0.70 – 1.29)	0.760	**-**	**-**
**Heard about FP from field health worker**				
No	1			
Yes	1.21 (0.90 – 1.63)	0.217	**-**	**-**
**Heard about FP at health facility**				
No	1			
Yes	1.09 (0.89 – 1.32)	0.400	**-**	**-**
**Heard about FP on mass media**				
No	1		1	
Yes	1.19 (0.96 – 1.48)	0.118	1.19 (0.95 – 1.48)	0.130
**Informed choice**				
No	1		1	
Yes	0.65 (0.53 – 0.79)	< 0.001	**0.59 (0.48 – 0.72)**	** < 0.001**

Also, women with four children [aOR, 2.18; 95% CI: 1.11 – 4.28] and five or more children [aOR, 3.62; 95% CI: 1.84 – 7.13] were more likely to experience a contraceptive failure compared to those with no child.

Women who had secondary education [aOR, 1.57; 95% CI: 1.04 – 2.35] and higher education [aOR, 1.84; 95% CI, 1.09 – 3.09] were more likely to discontinue contraceptives due to failure compared to those who had no formal education. Women who currently in union/living with a man [aOR, 1.77; 95% CI: 1.04 – 3.01] were more likely to discontinue contraceptives due to failure compared to those who had never been in union.

Women who had an informed choice on contraceptives were less likely to discontinue contraceptives due to failure [aOR, 0.59; 95% CI: 0.49 – 0.72] compared to those who did not have an informed choice (Table 4).

## Discussion

Contraceptive discontinuations contribute sustainably to the total fertility rate, unwanted pregnancies, and induced abortions [25]. This study aimed to establish and describe the prevalence of discontinuation of contraceptives due to failure among women aged 14–49 years in Uganda. Overall, the prevalence of failure-related discontinuation among Ugandan women was (5.6%) and varied by age, type of place of residence, phone ownership, access to mass media, Level of education, and marital status. We also found failure-related discontinuation to be strongly associated with the type of contraceptive method used [33].

In this study, women with access to the internet and mass media had high failure-related contraceptive discontinuation. This could be because women with access to the internet and mass media can easily access information on the different contraceptive methods and are, therefore, more likely to stop or switch to better contraceptive methods when they experience side effects [34].

Failure-related contraceptive discontinuation by methods were high in women who used traditional methods than in those who used modern contraceptive methods [35–37]. For modern contraceptive methods, women using short-term methods like pills and Injectables were more likely to experience failure than those using long-term contraceptive methods. This could be because of inconsistency when using short-term contraceptive methods, which expose women to increased failure rates. Similar findings were reported in [9, 33, 38]. Thus, information and counseling should be emphasized for short-term method users to prevent failure.

In multivariable analysis, the most important factors associated with contraceptive failure were women's age and informed choice. Women who were provided with adequate information about a particular contraceptive method and given an opportunity to make an informed choice were less likely to experience failure-related contraceptive discontinuation than women who did not make an informed choice. This could be because a woman who made an informed choice had adequate information about the contraceptive method of her choice and was able to effectively deal with its side effects without necessarily halting contraceptive use [39, 40]. This finding suggests that more time should be dedicated by clinicians to providing information on contraceptives to women before use. Furthermore, Women above 40 years had lower failure-related contraceptive discontinuation than women of other age brackets. This could be because women above 40 years are more likely than younger women to desire a permanent form of contraception [41, 42]. This could also be explained by lower fecundity, less frequent sexual intercourse, and higher compliance with contraceptive regimens, as evidenced by previous studies [43].

Lastly, multivariable analysis also showed that women with a higher level of education, five or more children and women who were currently in union/living with a man were more likely to experience failure related contraceptive discontinuation. This findings are in agreement with previous studies on contraceptive discontinuation in different countries [32, 33, 44–47].

We acknowledge that there are other reasons for discontinuing contraceptives, such as partner refusal, side effects, and method unavailability [9, 33, 48].

This study used retrospective data from the DHS. The DHS follows a rigorous methodology employing a two-stage cluster randomized sampling. We used sample weights to provide estimates reported in this study. However, we acknowledge that contraceptive failure could best be studied using cohort studies. Additionally, because the replies are self-reported and cannot be verified, there is a risk of information bias due to the sensitive nature of sexually related topics. But this study provides further information about the current state of the prevalence of discontinuation of contraceptives due to failure among women of reproductive age in Uganda.

## Conclusion

Our study found that there was a significant prevalence of discontinuation of contraceptives due to failure among women of reproductive age in Uganda and that it varied by women's age, level of education, exposure to the internet and mass media, and informed choice. These findings highlight the need for improved counseling services and contraceptive quality to help women and couples use methods correctly and consistently.

## Data Availability

The datasets generated and/or analyzed during the current study are available in the Demographic and Health Survey program website, https://dhsprogram.com/data/available-datasets.cfm and can be accessed after obtaining approval.
